# Characterization of Shallow Ground in Railway Embankments Using Surface Waves Measured by Dark Fiber Optics Sensors: A Case Study

**DOI:** 10.3390/s23239397

**Published:** 2023-11-25

**Authors:** Edwin Obando Hernandez, Paul Hölscher, Pieter Doornenbal, Cees-jan Mas, Joost van ‘t Schip, Agnes van Uitert

**Affiliations:** 1 Deltares, 2600 MH Delft, The Netherlands; paul.holscher@deltares.nl (P.H.); pieter.doornenbal@deltares.nl (P.D.); 2 ProRail, Moreelsepark 3, 3511 EP Utrecht, The Netherlands; ceesjan.mas@prorail.nl (C.-j.M.); agnes.vanuitert@prorail.nl (A.v.U.)

**Keywords:** fiber optics, dark fiber, surface waves, critical train speed

## Abstract

For the maintenance of railways on soft soils, accurate knowledge of the subsoil conditions is essential. Soft soils at shallow depths have high variability; thus, high spatial resolution is required. Spare telecommunication fiber-optic cables, known as dark fiber, can be used as an array of sensors to measure waves generated by running trains, which offers a unique opportunity to characterize shallow soils at high spatial resolution. We used dark fiber to measure seismic waves generated by running trains and implemented a seismic interferometry technique to retrieve surface waves. We evaluated the reliability of selected parts of the recorded signals split as *bow* waves (the train approaching the fiber), *train* waves (the train passing alongside the fiber), and *tail* waves (the train leaving the fiber) to retrieve broad-band surface waves. The analysis was performed in two distinctive zones. Zone I consists of a thick–soft (2.0–6.0 m thickness) layer, and Zone II consists of a thin–soft (less than 2.0 m thickness) layer, both overlaying a “stiffer” sand layer. At Zone I, *train* waves yielded the best results in revealing the thick–soft layer. At Zone II, the *bow* waves yielded clear high-frequency energy, revealing the overall soil structure but without identifying the shallow thin–soft layer.

## 1. Introduction

DAS (Distributed Acoustic Sensing) technology that uses fiber-optic cables as seismic sensors has emerged as a useful and promising tool for geophysical surveying and monitoring. DAS offers the opportunity to perform seismic measurements with unprecedented high spatiotemporal resolution and at a potentially low cost. Recent developments in DAS have also demonstrated its value in retrieving relevant information in various applications, such as geothermal well monitoring [1,2,3], earthquake monitoring [4,5], and ground characterization via surface wave surveying, using tailor-made arrays [6].

For railway applications, fiber-optic measurements have been successfully implemented for train positioning, speed monitoring, rail track health monitoring, and roadbed velocity imagining [7]. Accurate measurements with very high spatial sampling have been obtained using Brillouin optical time domain analysis (BOTDA), as well as optical frequency domain reflectometry (OFDR). These techniques, however, suffer from a lack of either monitoring capabilities or limited distance coverage, which represents a limitation when monitoring large railway lines is required. DAS, on the other hand, offers excellent monitoring capabilities at very long fiber optic cables with excellent dynamic responses and sensitivity to various types of sources [7]. DAS has been successfully implemented in detecting third-party interference, digging, and theft activities. It also serves as a tool to monitor the conditions of the wheels of the train, which causes a characteristic wave signature when there are flat-wheel problems [8,9]. Most of the DAS interrogators available offer high spatiotemporal resolution at very large distances up to 50–70 km. The strain sensitivity is in the order of 10^−9^ and a minimum gauge length between 1–2 m [7], which offers the possibility of studying railway-infrastructure-related problems at various scales.

Existing massive fiber-optic telecommunication networks containing hundreds of kilometers of abandoned/spare fibers, also known as dark fiber [10,11], are available everywhere. These networks can be potentially used as a large seismic array for survey and monitoring purposes. Surface waves can be generated by a variety of sources, including earthquakes, explosions, and vibrations from human activities such as traffic and construction. In the context of railways, surface waves are generated by running trains, which can be utilized to extract valuable information about the conditions of the ground underneath the embankment structure [12,13]. Surface waves can be effectively used to extract elastic properties in terms of S-wave profiles along the railway line, often required for maintenance purposes.

Surface waves generated by passing trains are extracted via the seismic interferometry method [14,15,16]. This method involves recording seismic waves at one location and using those recordings to generate synthetic seismic data at another location [16]. The basic idea behind seismic interferometry is to use the wavefield recorded at a receiver to simulate the wavefield that would have been recorded at another receiver. This is achieved by using the recorded data to create a virtual source, which emits waves that travel through the subsurface and arrive at the second receiver location. This procedure can be implemented in a series of receivers deployed in a linear fashion enabling the reconstruction of a shotgather, similar to the one retrieved via a hammer blow impact.

The use of running trains as a seismic source to retrieve surface waves was previously implemented by using standard geophone arrays [12] and, more recently, dark fiber-optic telecommunication cables as a seismic sensor array [10,11]. It has been demonstrated that the records generated after combining approaching and leaving train signals enhance surface waves suitable for subsurface ground characterization [11]. However, the influence of the signals utilized to retrieve the surface waves, created by either an approaching, passing, or leaving train, in relation to the soil conditions has not been investigated.

In this research, we investigated which parts of the waves generated by running trains are better suited to retrieve broad-band surface waves for variable soil conditions along the survey line. We performed a field test along a railway segment of 5 km at the railway between Zwolle and Lelystad in the Netherlands (from now on referred to as the Hanzelijn corridor). The measurements were performed using a telecommunication dark fiber-optic cable sensor alongside the railroad. The dark fiber cable was embedded in two distinctive soil conditions. The measured dark fiber records were grouped into three datasets defined as *bow*, *train*, and *tail* waves. *Bow* waves are those signals generated by the approaching train, just before the train signal amplitude is observed in the selected fiber-optic segment. *Train* waves are waves generated by the actual train passing alongside the selected fiber-optic segment. *Tail* waves are the waves created once the train has left the selected segment. The three datasets were processed separately using the seismic interferometry method, aimed at retrieving virtual shotgathers that contain surface waves.

The results presented in this work are aimed at improving the knowledge of how to maximize the benefits of the energy generated by running trains, which leads to high-resolution S-wave profiles at shallow depths underneath railway structures.

## 2. Site Conditions at Hanzelijn

**Figure 1:**
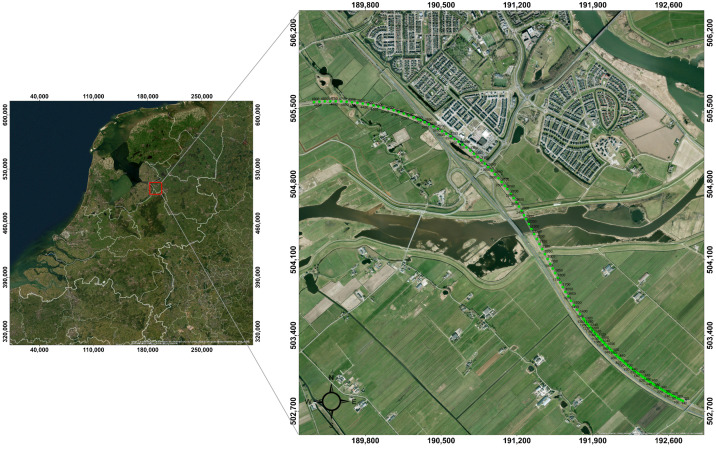
Location map of the Hanzelijn corridor. The verified coordinates of the fiber-optic cable channels alongside the railroad are indicated by green dots. The first channel is 120, which is the one closest to the measuring station localized at the intersection at the southeast side of the dark fiber-optic array.

The Hanzelijn corridor is localized west of Zwolle in the province of Overijsel in the Netherlands (Figure 1). The projection of the dark fiber-optic cable alongside the railroad line is indicated by green dots. The geology of the Netherlands consists mainly of sedimentary deposits from the Cratonian to the Quaternary. Pleistocene sand deposits and Holocene marine and fluvial deposits dominate. For the Hanzelijn corridor, the lithological composition and genesis of this part of the subsurface relevant for geotechnical constructions are presented here from old to young [17]. The Pleistocene deposits mainly consist of sand originating from a former Rhine system, cover sands formed in the latest ice age, and/or small-scale stream deposits. During the Holocene, with higher sea levels and, thus, higher groundwater levels, peat could grow on top of these sandy formations, alternating with clay deposits.

The geology and geotechnical conditions of the Hanzelijn corridor were carefully assessed during the construction of the railways (2006–2012) [18]. These investigations included cone penetration tests, boreholes, geophysical surveys, and laboratory tests. The results of these investigations were used to design and optimize the foundations, embankments, and cuttings of the railway line to ensure its stability and safety. Existing reference surface-wave measurements [19] carried out between channels 120 and 220 indicate that the soft-soil formation from the top surface down to a –5 m depth (including the manmade sand body) varies between 80 and 115 m/s. Deeper than –5 m, the S-wave velocities go higher than 250 m/s. This information will be very useful in interpreting the DAS results at the thick–soft soil conditions.

**Figure 2:**
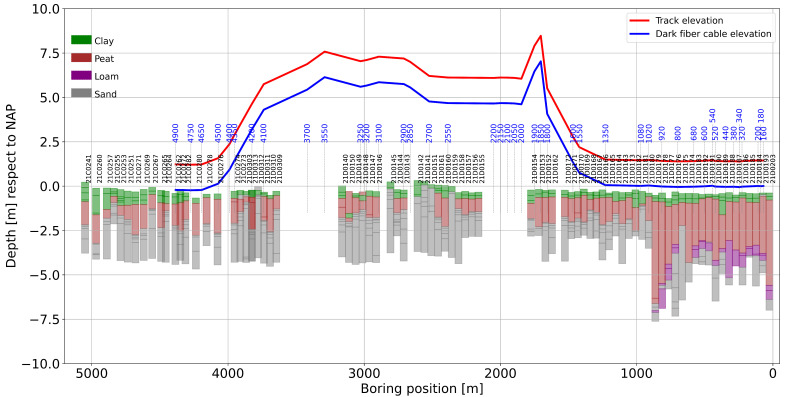
Lithological cross-section along the 5 km segment. The blue line corresponds to the approximate elevation of the fiber-optic cable, and the red line is the elevation of the track. The blue text is the first channel number of the 100-channel records utilized for the analysis. Fiber-optic channel numbers on the right side in blue (the color that is not as visible) correspond to channels 160, 180, and 200. Notice that the horizontal axes are plotted right to left for better comparison with the location map of Figure 1.

In Figure 2, the lithology cross-section along the survey line is plotted together with the selected dark fiber-optic segments, indicated by blue-colored numbers (the numbers correspond to the first channel of the selected 100-channel fiber segments). The elevation of the fiber-optic cable (blue line) with respect to the track position (red line) is also indicated. Notice that the fiber-optic cables are buried about 0.6 m from the free surface. All elevations are relative to the NAP (Normaal Amsterdams Peil) reference, which is the reference elevation for the Netherlands.

Alongside the fiber-optic segment, the lithology consists of soft sediments constituted by clay and till of variable thickness, overlaying a consolidated sand formation. Along the dark fiber-optic line, a thick–soft sediment deposit varies from 4 m to 6 m thick, which is predominantly southeast between channels 160 and 920 (see the lithology displayed in Figure 2). After channel 920, there is a predominance of stiffer soils with a thin–soft sediment of 2.5 m, which becomes thinner toward the northwest. Notice the difference in elevation between the track and the boreholes, especially between 1600 and 4500. The borehole logging was made before the construction of the railway. Then, at this location, a 7 m high embankment was constructed in order to cross the Reeve Diep and the infrastructure near the town of Kampen. Thus, for interpretation, only the first 1600 m will be utilized, especially for the inversion, where the prior knowledge of the sediment/sand transition will be utilized to define the search space to increase the accuracy of the computed S-wave velocities. For now, fiber-optic segments 160–1020 are referred to as Zone I, and segments 1020–4900 are referred to as Zone II. During the construction of the railway, an embankment was built by using sand. The original surface of this area is about −0.5 m NAP in Zone I to 0.0 m NAP in Zone II, as is visible in Figure 2. Considering that the embankment will be settled because of the compression of the soft clay and peat layers, the embankment has a thickness of more than 2 m to 7 m at the crossing of the water, the Reeve Diep (2000 m), and infrastructure (3700 m).

## 3. Seismic Interferometry

Seismic interferometry is a technique utilized to extract information (elastic properties) from the ground subsurface by processing spatially distributed vibration data, generated for this purpose or via ambient noise. The seismic interferometry method has become a very popular tool for constructing active-like surface-wave shotgathers utilizing low-frequency surface waves [14]. The basic principle behind seismic interferometry is that the seismic waves recorded at one location can be used as a source to create a virtual seismic record at another location. This is achieved by cross-correlating the seismic waveforms recorded at the two locations.

Following the formulation by Wapenaar [16], for two seismic stations, $x_{A}$ and $x_{B}$, the cross-correlation function, $G\left(x_{B},x_{A},t\right)$, can be defined as

(1) $$G\left(x_{B},x_{A},t\right)=G\left(x_{B},x_{s},t\right)*G\left(x_{A},x_{s},-t\right)$$

where *G* stands for Green’s function. The asterisk represents temporal convolution, and the time reversal of Green’s function turns convolution into a correlation. This formulation represents the cross-correlation of two receivers and provides the response at one of these receivers assuming that there is a source at the other receiver.

Thus, applying this principle to consecutive receivers, it is possible to construct pulse-like shotgathers similar to an active hammer-blow shotgather, which is referred to as a virtual shotgather. It has been demonstrated that when using non-standard seismic sensors, such as DAS sensors, seismic interferometry provides meaningful results comparable to standard sensors. DAS has been successfully implemented for shallow seismic characterization in urban environments using traffic noise [15]. The advantage of the large number of channels provided by DAS processed via seismic interferometry is that it allows us to create virtual seismic records at locations where we may not have been able to place a physical receiver or geophone because of space accessibility, which requires less labor and, hence, fewer resources.

Virtual shotgathers computed via seismic interferometry can be utilized to calculate the phase velocity spectrum using standard wavefield transformation methods, such as the MASW (Multichannel Analysis of Surface Waves) method developed by Gabriels [20] and popularized by Park [21,22,23]. The MASW method is widely used in geotechnical and engineering applications to obtain information about the subsurface shear wave velocity structure. The computed phase velocity spectrum is used to extract the dispersion curve from the fundamental mode (and higher modes if present), which is the input used to calculate the S-wave velocity structure via inversion.

The interpretation of the extracted measured dispersion curve is focused on determining the minimum and maximum wavelengths that can be realistically determined. The wavelength is defined as the ratio between phase velocity and frequency. The high-frequency limit (the short wavelength) provides information on the shallowest layer thickness to be revealed, while the low-frequency limit (the long wavelength) defines the maximum depth of exploration. As a rule of thumb, we adopt the criteria proposed in the literature [24] that suggest that the minimum and maximum depths of exploration are equivalent to one-third of the minimum and maximum measured wavelengths, respectively.

## 4. Data Acquisition

### 4.1. Acquisition Setup

**Figure 3:**
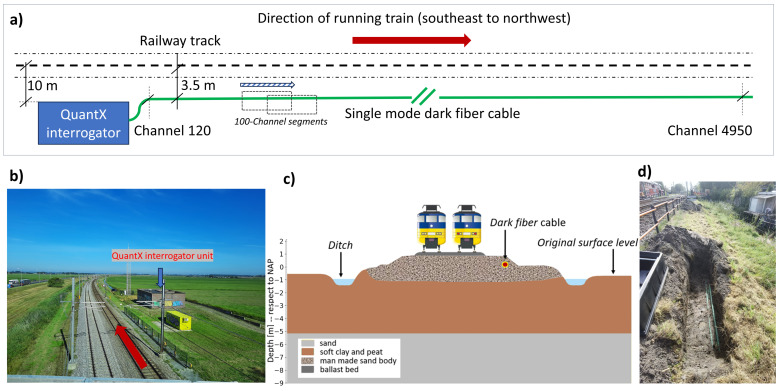
(**a**) Block diagram of the dark fiber setup utilized for data acquisition. The diagram illustrates the dark fiber cable position with respect to the railway track. The 100-channel segments were selected for processing after the data were recorded. (**b**) Panoramic view of the measuring station at Henzelijn corridor. (**c**) Cross-section of the embankment structure and fiber optic position. (**d**) Example of an HDPE pipe that contains the fiber alongside the railways at the Hanzelijn corridor.

The acquisition setup consists of a dark fiber (a spare single-mode telecommunication fiber-optic cable) of about 5 km in length utilized as a multichannel seismic sensor array. A scheme of the dark fiber cable alongside the railway is displayed in Figure 3. Figure 3a shows the positioning of the dark fiber cable placed at about 3.5 m from the middle of the railway track, where trains travel from southeast to northwest. To reach high spatial resolution, train passage signals should be recorded every 1.0 m along the whole 5 km of the railway. For this purpose, the DAS interrogator assigns channels along the dark fiber cable (simulating a multi-sensor array) separated by every 1.0 m. The first DAS channel that is close to the measuring station is channel 120, and the one at the end of the dark fiber cable is channel 4950. The measuring station, displayed in Figure 3b, is localized at about 10 m from the railway. The thick dark-red arrow indicates the railway track utilized for trains traveling southeast to northwest (the same as in Figure 3a). Figure 3c shows the cross-section near the measuring station (the soft sediment layer in Zone I). The cross-section shows the soil layer underneath the rail track, including the ballast bed, and the manmade sand body. The dark fiber-optic cable is embedded at about a 0.6 m depth inside the shallow man-made sand body or the embankment, as indicated by the red dot.

The dark fiber cable is packed in an HDPE (High-Density Polyethylene) pipe alongside the railway track (Figure 3d). It is important to notice that the HDPE pipe that contains the fiber appears to be very well coupled to the ground, which enhances the quality of the recorded signals.

### 4.2. Recording Parameters

**Figure 4:**
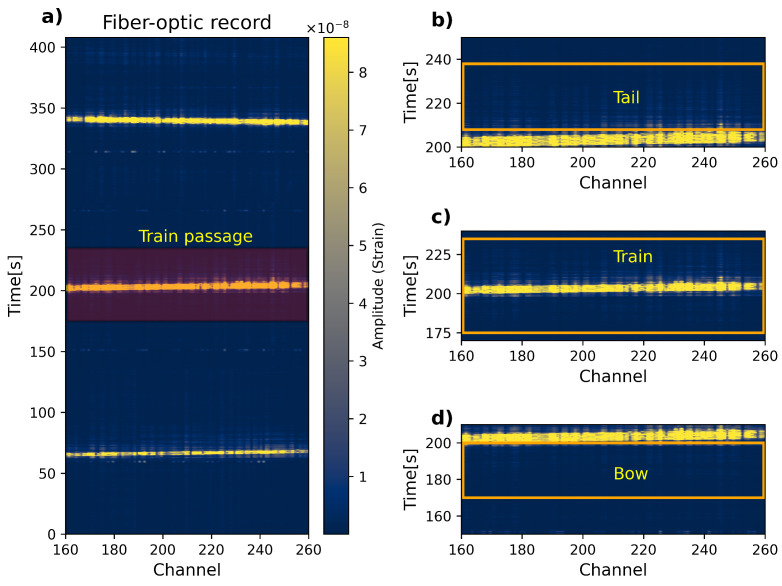
(**a**) Example of a dark fiber-optic record that contains 3 train passages of 100 signals (between channels 160 and 260). The plot also highlights the (**b**) *tail*, (**c**) *train*, and (**d**) *bow* wave signals utilized for later processing.

Data recording was performed using the DAS interrogator QUANTX manufactured by OptaSense a LUNA company based in, London, the UK (United Kingdom). It is an ultra-high-performance quantitative distributed Rayleigh sensor. The system offers a user-selectable gauge length option between 2.0 m and 205.4 m and output channel spacing between 1.0 m and 10.27 m. It measures a maximum fiber length of 200 km and has a maximum interrogation rate of 10 kHz. For this project, data recording was performed using a sampling frequency of 5 kHz divided by a factor of four, providing output signals of 1.25 kHz. This output sampling frequency enables a maximum usable frequency of 625 Hz. The channel spacing was set to 1.0 m, and to achieve the highest possible spatial resolution at an acceptable SNR, the gauge length was set to 2.0 m. These settings provide high-spatial-resolution data that should yield comparable results to standard linear geophone arrays utilized in active surface-wave surveys [25]. The sampling frequency and channel spacing determined a maximum measurement length of 5 km. For 5 km of track, the dark fiber-optic sensor allowed for up to 5000 channels. The aim of the measurements was to record the seismic waves generated by train passages. Thus, the system was set to continuous recording mode (no threshold limit was utilized) to pick up the highest possible number of trains running along the 5 km track segment during the busiest hours of the day. The monitoring campaign was conducted by ProRail for over 2 weeks. The recorded data were saved in sections of 120 s containing 5000 channels. After the measurements, segments of 100 channels were selected at various locations (consecutive segments can be selected with some overlapping channels, as indicated in Figure 3a) to be later processed and to extract the local soil properties. An example of a selected dark fiber record of 100 signals (delimited by channels 160–260) that contains three train passages is displayed in Figure 4a.

To describe the type of waves utilized for our analysis, in Figure 4a, we highlight (semitransparent dark-red background) a single train passage. Figure 4b shows *tail waves* that depict 30 s after the train crosses the fiber segment. Figure 4c shows *train waves* that depict 60 s containing the full train passage signal in the middle. Figure 4d depicts *bow waves* that depict 30 s before the train signal appears. The example displayed in Figure 4 corresponds to a selected dark fiber segment bounded by channels 160–260, but other fiber segments can be selected (with some overlap), as displayed in the block diagram shown in Figure 3a. For processing, only those records with trains traveling in a southeast-to-northwest direction are utilized. Likewise, fiber segments over bridge infrastructures where the dark fiber was not coupled to the ground are also discarded.

## 5. Processing Scheme

In this section, we describe the processing scheme adopted. We distinguish data pre-processing; seismic interferometry; dispersion analysis; and finally, the S-wave velocity computation.

In standard active MASW processing, the sources are located at a certain distance from the measurement array and can be considered to be at a fixed position. By using a train as a vibration source, the source is considered “fast”, moving inside the measurement array. This leads to waves running in two directions in the array, which generates different types of waves depending on the positions of the moving source. To assess the reliability of waves generated by a passing train in capturing surface waves that can be used to characterize the shear wave velocities underneath, the data are processed separately, for *bow*, *train*, and *tail waves*. Thus, the processing scheme described in the upcoming sections is the same implementation for each dataset.

### 5.1. Data Preprocessing

The data were recorded in the native format HDF5 (Hierarchical Data Format, Version 5) and stored in time segments of 120 s. As the target records are those where there is a train passage, a selection procedure was implemented to identify either full or partial train signals. Before running the selection, the signals are bandpass-filtered between 1.0 and 100.0 Hz. The selection process is performed for those busy hours between 6:00 and 22:00. The stored data are used to refine the selection, which involves splitting the dataset into *bow*, *train*, and *tail* waves, as shown in Figure 4. The fiber-optic data are split into segments of 100 channels that cover 99 m. The 100-channel segments were created every 20 m inside Zone I and every 50 m inside Zone II (see sliding window over the fiber line with overlap in Figure 3a). The segments are named according to the number of the first channel that constitutes the 100-channel record.

Prior to data processing, the preparation of the input dataset was carried out as follows:

- Train signal identification and selection recorded over several days. We identified about 60 train passages per day. These trains were all running southeast to northwest.
- Storing single passages of 100 channels of 120 s. The train signals are in the middle of the 120-s record.
- Splitting 120-s signals into records of 30 s before (*bow* waves), 60 s during (*train* waves in the middle of the record), and 30 s after (*tail* waves) the train passage.
- Concatenating individual 30 s records to create 30 min single records.
- Assembled records of 30 min (60 train passages) are stored in the *.sac format for the *bow* and *tail* wave datasets. For *train* waves, the total duration of the assembled records is 60 min.

Notice that the fiber segments are named using the first channel number, as shown in Figure 1.

### 5.2. Seismic Interferometry

Once the input data are ready, seismic interferometry is carried out using the standard procedure suggested by Bensen [14], successfully implemented in previous works [10,11].

Thus, the processing is implemented as follows:

- Time domain normalization using one-bit normalization.
- Frequency domain normalization and spectral whitening.
- Cross-correlation using a time lag of 10 s.
- Band-filtering of cross-correlated signals [1.0–50 Hz].
- Phase-weighted stacking (PWS) of cross-correlated signals over 30 min.

The seismic interferometry processing is implemented using the NoisePy tool (version 1.0) developed by Jiang and Denolle [26]. As mentioned, the computation is carried out separately for the *bow*, *train*, and *tail* wave datasets and also stored separately. The source–receiver corresponds to the channel number used to name each dark fiber segment (number in blue shown in Figure 2). Considering the highly repetitive nature of the train-related signals, most of the selected segments (only a few segments were stacked over 2.0–2.5 h of the assembled signals or 240–300 train passages) are processed using 60 train passages, which appeared to be enough to have a clear and stable phase velocity spectrum.

### 5.3. Dispersion Analysis

The dispersion analysis consists of computing the phase velocity spectrum using the active-like MASW scheme [25] for virtual shotgathers computed from the bow, train, and tail wave cross-correlated functions. For dispersion processing, the frequency range is set between 1 and 30 Hz. The computed spectrum serves as a parameter to evaluate the performance of the three types of records (*bow*, *train*, and *tail*) analyzed at Zone I and Zone II.

The computed spectrum will provide the dispersion curves of the fundamental mode (and higher modes when present) required as input to compute S-wave velocity profiles at each selected site. For the final selection of the dispersion curves, we use the spectra (from the bow, train, and tail datasets) that depict clear dispersion trends.

### 5.4. S-Wave Velocity Computation

The 1D S-wave velocity profiles are computed by inverting measured dispersion curves at all analyzed locations. The inversion is carried out using a stochastic search-based algorithm developed by Wathelet [27] coded with the *Dinver* tool from Geopsy. Consecutive inverted 1D S-wave profiles are used to display an S-wave velocity cross-section alongside the fiber-optic line, which serve as a general assessment/overview of the stiffness variation underneath the fiber-optic line.

## 6. Results

This section starts with a discussion of the frequency content of the recorded signals along the dark fiber-optic sensors in relation to the actual geological conditions of the site. Then, we describe the apparent velocities observed in the frequency–wavenumber spectrum for two selected sites, localized at the thick–soft (Zone I) and thin–soft to stiff soil (Zone II) conditions. Later, we focus on the dispersion analysis at two of the same sites in Zone I and Zone II for *bow*, *train*, and *tail* waves. We also discuss the variability of the wavelengths measured along the whole dark fiber segments retrieved from the phase velocity spectrum. We continue with the inversion procedure implemented to retrieve the S-wave velocity profile at each selected segment, and finally, we present the 2D distribution of the S-wave velocities along the full dark fiber-optic cable.

### 6.1. Frequency Analysis

A first inspection of the recorded dark fiber data is made by characterizing the frequency response of the transient signals recorded at two selected locations within Zone I and Zone II.

**Figure 5:**
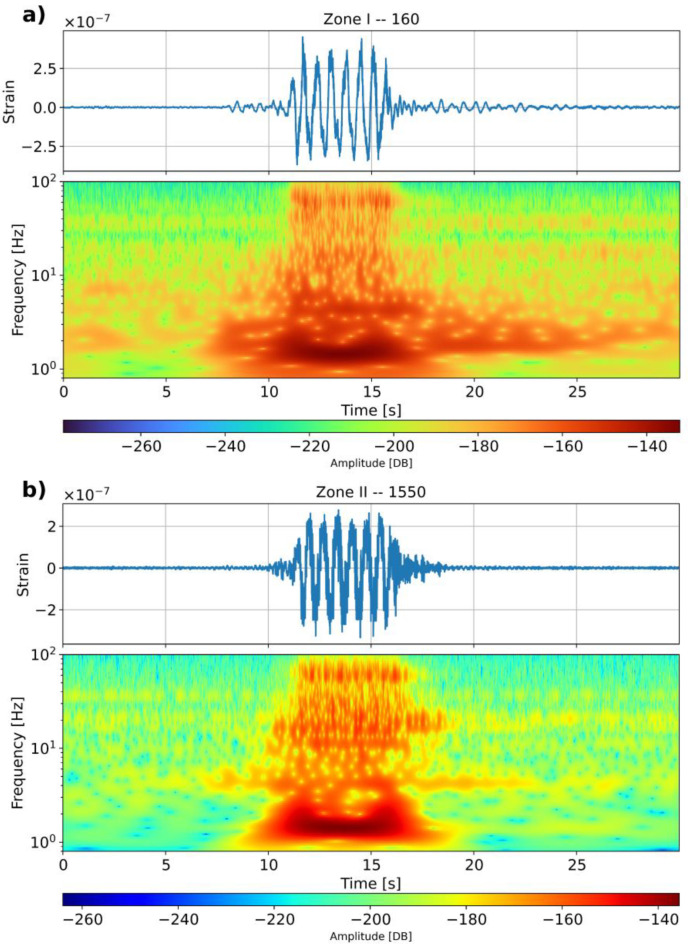
Single signal (**top**) and its spectrogram (**bottom**) for two full train passages recorded at (**a**) position 160—Zone I and (**b**) position 1550—Zone II.

We plot the typical train signal together with its spectrogram for waves recorded at channel 160 at Zone I (Figure 5a) and channel 1550 at Zone II (Figure 5b). It appears that at both Zones I and II, the recorded waveforms capture the dominant low-frequency components generated by the vibration of the train’s passage, characterized by eight low-frequency cycles. Zone II, however, seems to retrieve higher-frequency energy compared with Zone I, which can also be observed in the energy distribution depicted by the spectrograms. It is interesting to note that, in Figure 5a, at this thick–soft soil site, the vibration content at 1–3 Hz is clearly visible after the train’s passage. Based on the average vibration distance and train speed, the passage of the vibration leads to a signal in a range of 1–3 Hz. The embankment seems to vibrate on the soft soil after the train passage with a low frequency for a relatively long duration. This feature is not observed in the signal retrieved at Zone II. Thus, these distinctive features are related to differences in the actual soil conditions at both positions.

**Figure 6:**
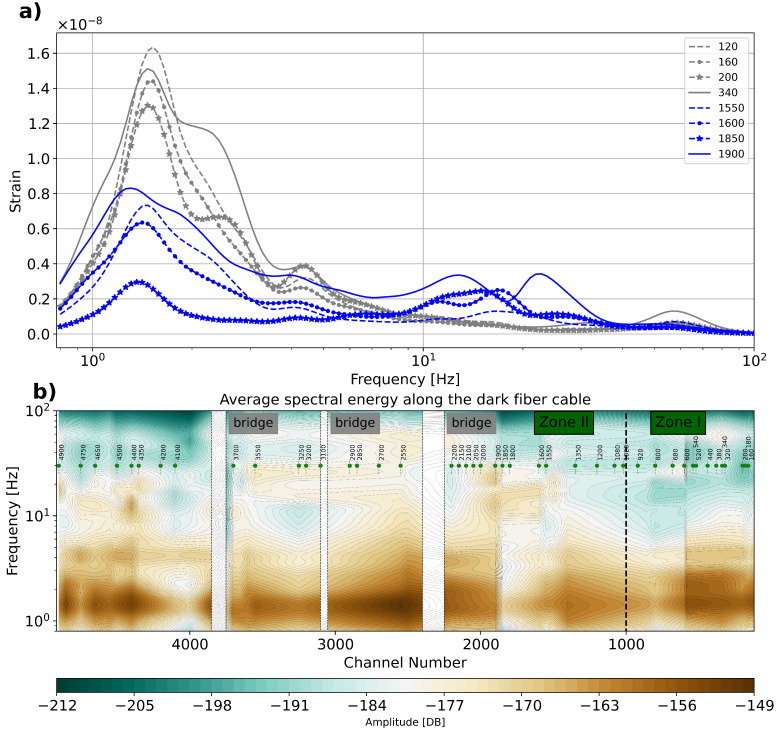
(**a**) Average spectrum for selected traces at positions in Zone I (120, 160, 200, and 340) and Zone II (1550, 1600, 1850, and 1900) and (**b**) energy distribution after interpolating individual average curves (frequency with respect to channel number). We also indicate the position of bridges along the fiber-optic line.

To assess the potential soil stiffness dependency of the recorded signals, we computed average spectra along various selected positions (Figure 6). In order to take advantage of the highly repetitive signals generated by a train passage, we used at least 60 train passages at various locations on the fiber line. Thus, we initially present (Figure 6a) the average amplitude spectrum for eight selected sites (four sites in each zone). It appears that in softer site conditions (Zone I), the low frequencies are dominant over the higher frequencies, while in stiffer site conditions (Zone II), the higher frequencies appear more pronounced. This result suggests that there is a clear difference in the system behavior of these two zones. The stiffer zone may be acting more as a half-space. Moreover, the absence of high-frequency energy in the soft site conditions may be caused by the higher attenuation induced by the very soft shallow layer.

We use the average spectrum to analyze the dependency of the spectral energy with respect to the soil stiffness conditions along the full fiber segment (Figure 6b). Thus, we use the spectra (like those displayed in Figure 6a) to construct frequency distributions for all analyzed positions (Figure 6b). From this plot, we recognize that, in Zone I (which was determined on the basis of the softer soils), the energy is concentrated everywhere in the low-frequency part and with almost no energy at the high-frequency part (above 15 Hz). On the other hand, in Zone II, we observe much more energy in the high-frequency part. We conclude that the distinctive variation in the energy distribution may be used as an indicator to determine the stiffness variation along the fiber.

### 6.2. Frequency–Wavenumber Spectrum

**Figure 7:**
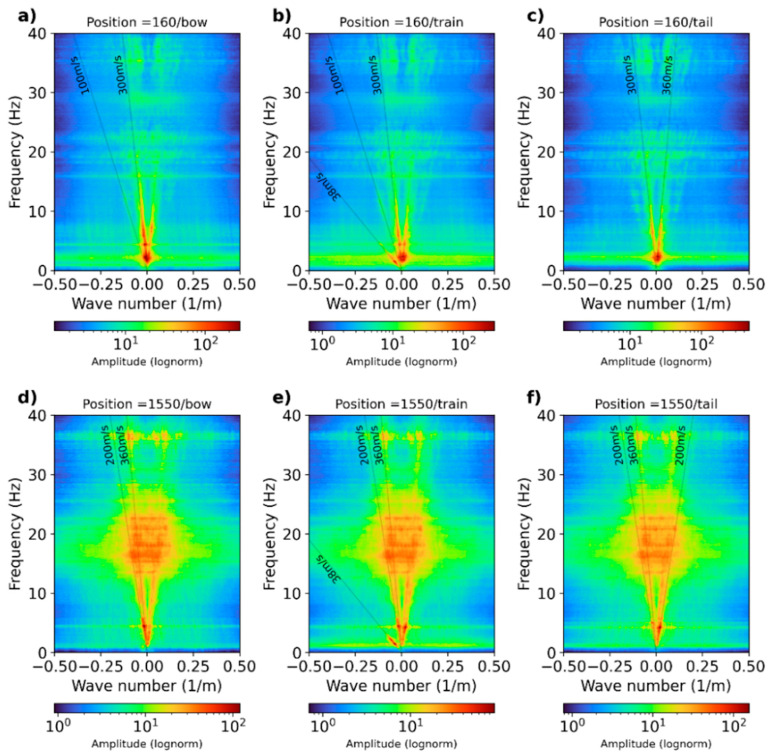
Stacked frequency–wavenumber spectra for (**a**–**c**) Zone I and (**d**–**f**) Zone II. The spectrum was computed after stacking individual f-k spectra at both selected locations.

A more in-depth analysis is performed by computing the frequency–wavenumber spectrum for selected dark fiber-optic records. We display the frequency–wavenumber or f-k spectrum for two selected positions at 160 (channels 160–260) and 1550 (channels 1550–1650) (Figure 7). We display the f-k spectrum for *bow* (Figure 7a,d), *train* (Figure 7b,e), and *tail* (Figure 7c,f) signals. The f-k spectrum was computed using at least 60 train passages.

Figure 7a–c show Zone I. The f-k spectrum shows consistent energy trends from 1 Hz to 20 Hz, which later become inconsistent at up to 40 Hz. The estimated apparent velocities are indicated by the thin line alongside each identified energy trend. For *bow* signals, Figure 7a displays a low-velocity term of about 100 m/s and a higher velocity of about 300 m/s. The 100 m/s apparent velocity is also present in Figure 7b for the *train* waves. The 300 m/s velocity is present in both the *train* and *tail* waves. A low-velocity energy of 38 m/s is dominant in Figure 7b, which is caused by the actual train passage. This aspect is visible in the *train* signals only. From these results, we interpret 100 m/s as the fundamental mode (M0) and 300 m/s as the first higher mode (M1).

Figure 7d–f show the f-k spectra for *bow*, *train*, and *tail* waves recorded at position 1550 in Zone II. Here, the apparent velocities appear higher than the ones measured at Zone I. The lowest velocity observed (38 m/s) appears to be identical to the one measured at Zone I, which occurs in the *train* wave dataset. The interpreted fundamental mode, M0, is 200 m/s, and the first mode, M1, is 360 m/s, and both are visible in the three types of signals.

Overall, the apparent velocities again appear higher in Zone II compared with the ones measured at Zone I, validating the idea that the retrieved signals will provide information regarding the actual elastic properties in the two distinctive soil conditions. Similar velocity values should also be observed in the phase velocity spectrum computed from the cross-correlated functions for the three types of datasets analyzed.

### 6.3. Dispersion Analysis

#### 6.3.1. Virtual Shotgathers

**Figure 8:**
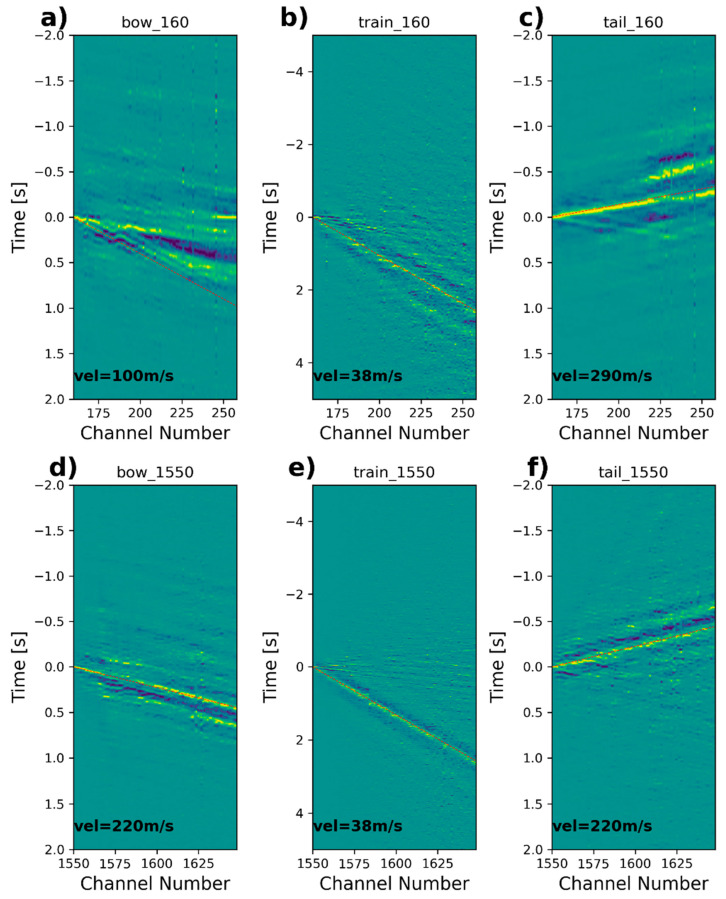
Computed virtual shotgathers from bow, train, and tail signal datasets for (**a**–**c**) Zone I and (**d**–**f**) Zone II.

Now, we display the results of the interferometry analysis, comprising the cross-correlated signals for the *bow*, *train*, and *tail* wave signals using fiber segments in Zone I and Zone II (Figure 8). We use the signals at segments bounded by channels 160–260 and 1550–1650 localized in Zone I and Zone II, respectively. We show both the positive and negative sides of the cross-correlation function. Notice that, in this section, we mainly describe the observed wave trends used to estimate the apparent velocity along the full 99 channels and discuss differences and similarities between the types of waves analyzed for both side conditions. More detailed variations in the actual velocities are described later on in the dispersion analysis.

The computed cross-correlations for Zone I are shown in Figure 8a–c. The virtual shotgathers calculated from the *tail* waves (Figure 8c) appear to be rather consistent along all traces concentrated on the negative side of the cross-correlation but with some wave trends on the positive side up to channel 220, with a dominant apparent velocity of about 290 m/s. The *bow* waves (Figure 8a), on the other hand, provided a slightly less consistent energy pattern with some apparent discontinuity bounded by channels 180–190. The *bow* waves also show more variability in apparent velocities with a dominant apparent velocity of about 100 m/s. The *tail* waves propagate in the opposite direction (Figure 8c). For *train* waves (Figure 8b), there seems to be a clearly dominant low-velocity trend of about 38 m/s, which propagates down to the whole fiber segment. This low frequency is associated with the actual train passage, which travels at the same low speed. Notice that, as expected, the wave speeds observed in the computed virtual shotgathers appear very similar to the apparent velocities observed in the f-k spectrum computed from the raw fiber optic signals.

The cross-correlation functions computed for Zone II are displayed in Figure 8d–f. The recovered surface waves appear, in general, rather coherent along the 100 traces with an apparent velocity of about 220 m/s, similar to the wave trend observed for the *tail* waves. When including the *train* waves, the dominant low-velocity trend of about 38 m/s is also present along the full fiber length. Notice that, this time, the *bow* waves (Figure 8d) seem to illuminate the whole 100-trace segment with higher velocities with respect to Zone I, validating the fact that the soil conditions are stiffer. The *tail* waves (Figure 8f) yield a similar wave trend but with less consistency/continuity compared with the *bow* waves. For *train* waves, again, there seems to be a dominant low-velocity trend clearly associated with the passing train, which acts as a moving source. In this zone, the faster waves running in both directions are softly visible in the virtual shotgathers.

#### 6.3.2. Phase Velocity Spectrum

Virtual shotgathers are used to compute the phase velocity spectrum at the two selected sites/segments localized at Zone I and Zone II. We describe the variations in the observed velocities retrieved in the phase velocity spectrum. In order to enhance the high-frequency part of the phase velocity calculation, we utilize the first 48 channels. The 48 channels target a theoretical maximum wavelength of 47 m, which yields a maximum exploration depth of about 16 m (one-third of the maximum measured wavelength).

**Figure 9:**
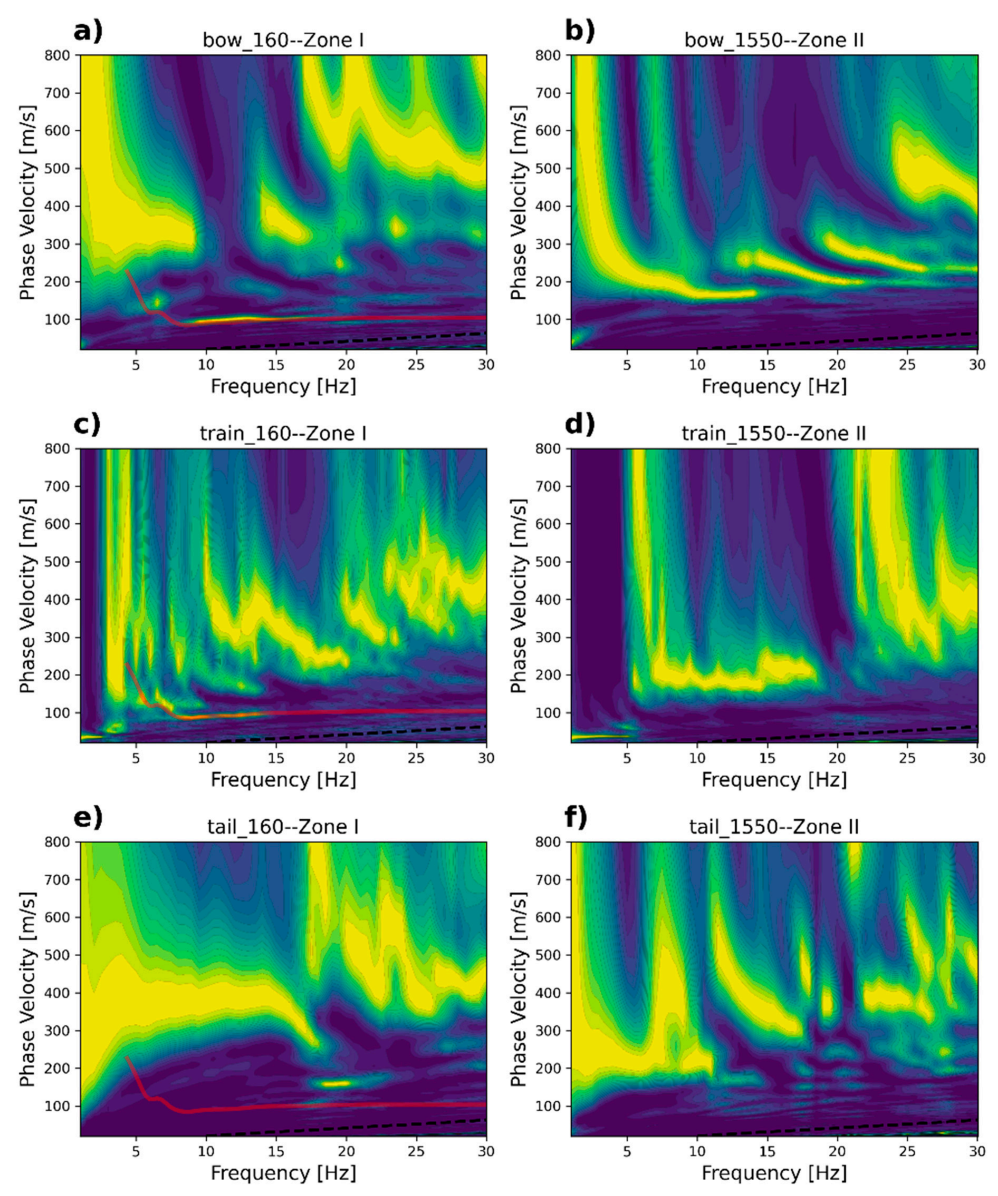
Phase velocity spectrum for (**a**,**c**,**e**) Zone I (160–260) and (**b**,**d**,**f**) Zone II (1550–1650). The black discontinuous line shows that the limit of the theoretical minimum wavelength in this case is 2.0 m. The red semi-transparent line represents the fundamental mode measured with 4.5 Hz geophones using 1.0 m separation localized on top of the fiber optic line at position 160.

Figure 9a,c,e show the spectra computed with *bow*, *train*, and *tail* waves located in Zone I. At this site, the characteristic dispersion curve measured with the standard geophone array and hammer blows is available [19]. The geophone array here was located on the free surface about 0.6 m above the estimated dark fiber-optic cable. The fundamental mode retrieved with standard geophones is delineated by the semi-transparent red line in Figure 9a,c,e. The low-velocity trends close to the 100 m/s trend retrieved with the *bow* (Figure 9a) and *train* (Figure 9c) waves appear to be in good agreement with the reference-measured dispersion curve. The *train* waves also show four less clear but identifiable higher-mode trends. Although the *bow* waves appear to be comparable to the reference dispersion curve at this site, this is not always as clear as the *train* waves along all other positions within Zone I. The spectrum computed with *tail* waves is displayed in Figure 9e. It appears that there is no energy at the low-frequency part, but it appears to be very prominent toward higher velocities between 300 and 400 m/s.

Despite the reasonable similarity between the dispersion trends of the bow and train waves with respect to the reference dispersion curve, in both cases, the energy occurs at a very limited frequency band between 4.5 and 17 Hz. This is almost half the frequency range observed with the standard geophone dispersion curve but enough to reach a minimum wavelength of about 5.89 m or a minimum exploration depth of about 1.96 m.

The phase velocity spectra for Zone II are displayed in Figure 9b,d,f. Figure 9b shows the dispersion trends of the *bow* waves. These appear much clearer compared with the ones retrieved from Zone I. The measured phase velocity of the interpreted fundamental mode varies from 170 m/s to 300 m/s in a frequency range between 4 and 20 Hz. There is also the presence of two clear higher modes, which propagate in a velocity range between 200 and 300 m/s up to a frequency of 30 Hz. Figure 9d,f show the phase velocity spectrum computed for the *train* and *tail* waves, respectively. Although both wave types show some multimodal patterns in a similar velocity range, with respect to bow waves, they are not well separated and, thus, difficult to interpret. This consistent behavior was observed in all segments analyzed along Zone II, indicating that, for this zone, the best results are obtained for *bow* waves or when a train is approaching.

### 6.4. Wavelength Coverage

For the maintenance and renewal of the railway embankment, knowledge of the thickness of the existing embankment and the soft soil layer between the embankment and the stiff deeper sand is required. Thus, determining the minimum exploration depth of the measured surface waves using dark fiber sensors is very relevant.

The minimum and maximum exploration depths are defined as one-third of the minimum and maximum measured wavelengths, respectively. In Zone I, the thickness of the soft soil sediment varies between 2 and 6 m. This means that the minimum wavelength must be about 6 m. In Zone II, the soft sediment layer is much thinner (thinner than 2.0 m). This leads to a shorter minimum wavelength, which is more challenging to determine. It is important to note that, in Zone II, the presence of the manmade layer between channels 1600 and 4500 prevents us from determining the thickness of the clay and the layer depicted in the lithological section, which was performed before the actual construction of the railroad section.

**Figure 10:**
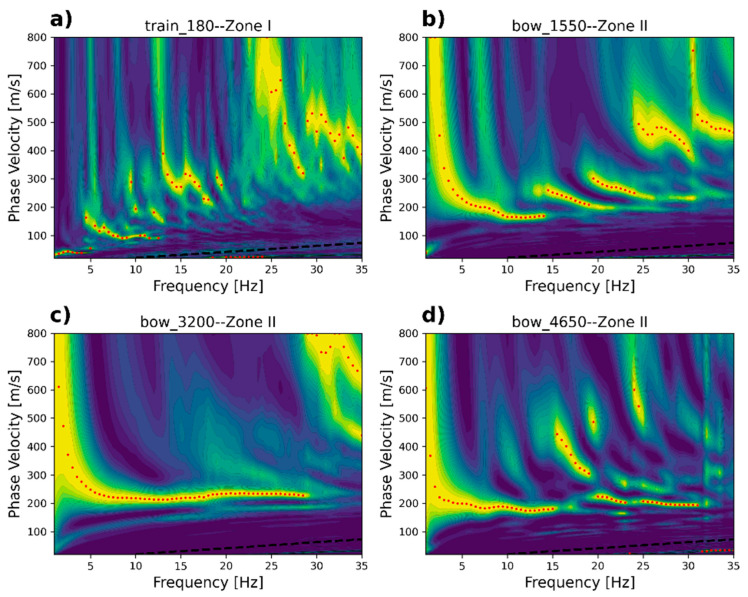
Phase velocity spectra distributed along the fiber-optic cable for Zones I and II at segments: (**a**) 180–228, (**b**) 1550–1598, (**c**) 3200–3248, and (**d**) 4650–4698. The black discontinuous line represents the limit of the theoretical minimum wavelength, 2.0 m. The red dots are the peak values of the dispersion trend, which serves as the basis for the interpretation of the dispersion curves.

We selected four dark fiber segments to display the variations in the measured surface-wave energy for various soil conditions. Figure 10 depicts the computed phase velocity spectrum (using 48 channels) for the thick–soft layer (160–1020), the thin layer (1020–1600 and 4500–4900), and the thick embankment (1600–4500).

Figure 10a displays the dispersion curve revealed at position 180 (channels 180–228). The fundamental mode occurs between 4.5 Hz (velocity = 180 m/s) and 13 Hz (velocity = 90 m/s). The minimum and maximum wavelengths are 6.92 m and 40.0 m, respectively.

Figure 10b shows a clear fundamental mode together with at least two well-defined higher modes. Although the peak values represented by the red dots are delineated by the main energy, the measured energy trends appear to be extended toward slightly higher frequencies. For example, the fundamental mode occurs between 4.5 Hz (velocity = 220 m/s) and 18 Hz (velocity = 190 m/s). The first higher mode occurs between 14.5 Hz (velocity = 260 m/s) and 30 Hz (velocity = 200 m/s). The second higher mode occurs between 18 Hz (velocity = 300 m/s) and 31 Hz (velocity = 225 m/s). Thus, based on the three energy trends interpreted, the minimum and maximum wavelengths are 6.7 m and 47.0 m, respectively, reaching minimum and maximum depths of 2.2 and 16 m, respectively.

Figure 10c shows a clear single-mode trend that varies between 5.0 Hz (velocity = 230 m/s) and 29.0 Hz (velocity = 255 m/s). In this location, the minimum and maximum wavelengths are 8.8 m and 46 m, respectively, which yield minimum and maximum exploration depths of 2.90 m and 15 m.

Finally, Figure 10d shows the clear low-velocity trend between 4.5 Hz (velocity = 200 m/s) and 23 Hz (velocity = 180 m/s). A second energy trend, interpreted as the first higher mode, occurs between 20 Hz (velocity = 220 m/s) and 32 Hz (velocity = 200 m/s). Thus, from the extracted frequencies and velocities, we can observe that the minimum and maximum wavelengths in this position are 6.25 m and 44.0 m, producing minimum and maximum exploration depths of 2.08 m and 15.0 m, respectively.

**Figure 11:**
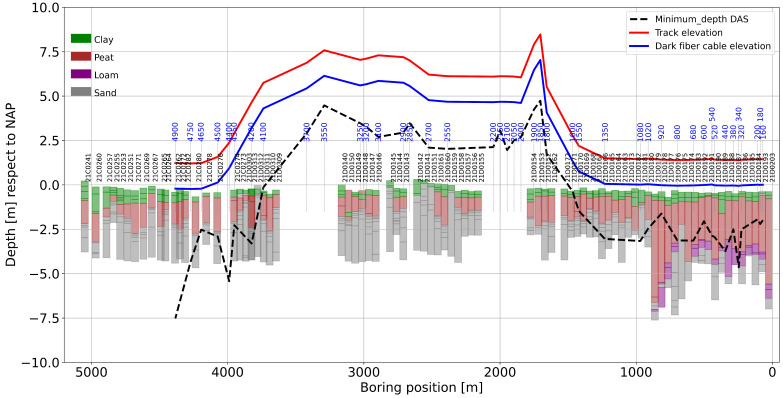
Minimum exploration depth with respect to fiber position, represented by the discontinuous line and determined using the minimum wavelength recorded. The blue line represents the elevation of the fiber-optic cable (buried about 0.6 m below the surface). The blue numbers represent the first channel (also used as a source channel) of the fiber-optic segments utilized for our analysis.

We performed a similar interpretation of all selected segments to determine the minimum inspection depths. Figure 11 shows that the computed minimum inspection depths were computed with respect to the fiber’s position in order to obtain an overview of what the resolution of the computed dispersion curves will be by using the dark fiber sensor at the Hanzelijn corridor and running trains as seismic sources. It also shows the position of the fiber and track together with the lithology profile. The value of this comparison is in defining to what extent the resolution provided by the interrogator and the energy retrieved from the source can detect the soft layers in Zone I and Zone II, represented by channels 160–1550.

In Zone I, the expected bottom of the embankment is above the inspection depth, and this cannot be observed (the first 2.0 m from the track elevation). The bottom of the soft soil layer (clay + peat/sand interface) is close to the minimum inspection depth. This indicates that we should be able to reveal the bottom of the soft and “thick” layer that characterizes Zone I (fiber segments 120–1000).

In Zone II, between segments 1000 and 1550, the minimum exploration depth goes deeper than the bottom of the soft soil (clay + peat/sand interface), so we cannot observe this. Between locations 1600 and 4200, the bottom of the embankment is under the inspection depth, so this interface might be visible. It is uncertain because of a lack of knowledge of the actual manmade layers. After fiber segment 4200, the minimum depth tends to be deeper than the actual clay and peat/sand interface.

### 6.5. Surface Wave Inversion

To characterize the S-wave velocity along the selected positions, we extracted the fundamental and higher modes (when present) from all computed phase velocity spectra, as shown in Figure 10. The extracted dispersion curves allow us to determine an overview of the S-wave velocity distribution along the 5 km fiber-optic segment. Notice that, for Zone I, where the elevation of the fiber positions is similar to that of the lithology, we used our knowledge of the clay + peat/sand interface to define the search space and depth boundaries to reduce the misfit of the inverted profiles. On the other hand, in Zone II, a priori information could not be incorporated (channels 1600–4500) given the presence of the thick embankment.

**Figure 12:**
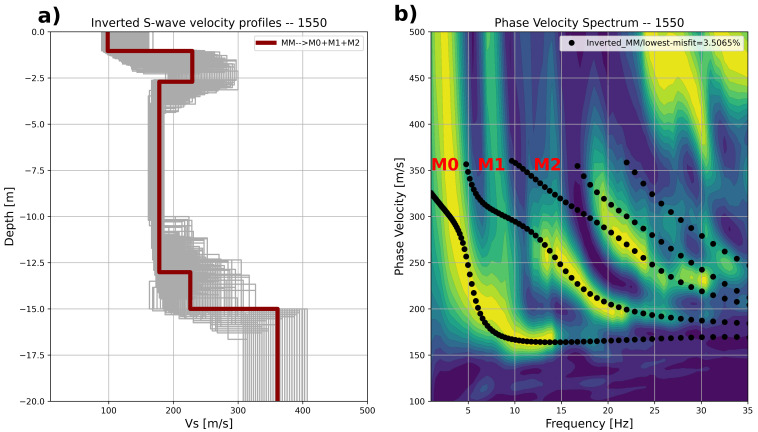
(**a**) Inverted S-wave velocity profile. The figure shows 1000 profiles; the best model is displayed in a dark-red color. (**b**) Phase velocity spectrum together with the forward response (black dots) of the best inverted model using the fundamental mode, M0; the first higher mode, M1; and the second higher mode, M2.

Figure 12 shows an example of one of the inverted S-wave velocity profiles displaying the S-wave velocity distribution at position 1550. This case was selected since it depicts very well-defined multimode dispersion trends. The inverted profiles for position 1550 are shown in Figure 12a. The forward response of the best inverted S-wave velocity profile (dark red line in Figure 12a) is plotted together with the interpreted modes (black dots) in Figure 12b. Notice that the lowest misfit of the best model is below 5.0%.

The location is at the ramp to the thick embankment, where the bottom of the soft layer is just around the inspection depth. The inverted model shows a top layer (~ first 2.5 m) with an S-wave velocity a bit higher than 200 m, which decreases down to 180 m/s. The main velocity contrast occurs at a −13.0 m depth. This shows that the best inverted model indeed provides a reasonable fit of the measured phase velocity spectrum.

The velocity profile in Figure 12a does not show a soft layer at the expected depth. Obviously, this soft soil layer is too thin (or too stiff) to be detected.

### 6.6. S-Wave Velocity Structure

**Figure 13:**
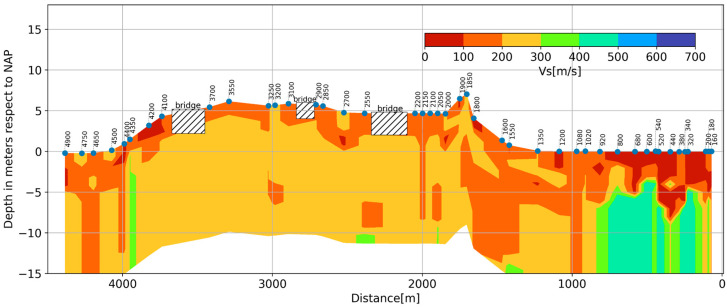
Interpolated S-wave velocity cross-section using individual inverted 1D S-wave profiles at selected locations. Segments with a bridge location are indicated by diagonal hatches. Fiber-optic channel numbers on the right side correspond to channels 160, 180, and 200.

Once all 1D S-wave velocity profiles are computed, they can be interpolated to describe the stiffness variation in terms of S-wave velocity (Figure 13). It appears that most of the variability occurs in Zone I (segments 160–1020), which is characterized by the soft–thick (clay and peat) layer that varies between 80 m/s and 110 m/s, overlaying a relatively stiff layer (sand) with an S-wave velocity of about ~400 m/s. The traditional geotechnical survey data were checked, and indeed, after channel 920, the properties of the deep layer changed.

In Zone II, there is less variation. Although the currently available method cannot deliver the thickness of the embankment and soft soil layers, it clearly provides information on the variability of the subsoil and positions where thick(er) soft soil layers are expected.

The 2D S-wave velocity cross-sections should be regarded as a general overview of the soil stiffness variation of the site. For detailed information about the selected segments, standard high-resolution surveys should be performed.

## 7. Discussion

The first assessment in the frequency domain shows that there seems to be a variation in the acoustic energy with respect to the soil stiffness conditions. This suggests that the soft soil condition enhances the low-frequency energy, associated with the traveling speed of the train. In the soft soil zone, the fiber did not capture many high-frequency vibrations that may be caused by attenuation induced by higher soil damping. In stiffer soil conditions, although the high-frequency energies are visible, the highest amplitudes still occur in the lower frequency range.

It is interesting to note that, in the shotgathers, the direction of the wave propagation (in the bow and tail signals) is preserved, but the potential effect induced by the moving nature of the source or the Doppler effect is not observed. It seems that the cross-correlation and the stacking procedure cancel the influence of the Doppler effect practically efficiently. This topic is not addressed in this research, but it will be interesting to investigate how this could interact with seismic interferometrical processing.

We examined the quality of the phase velocity spectra for *bow*, *train*, and *tail* time windows in two distinctive soil conditions. For the first 900 m—the site that is characterized by a 2–6 m soft soil layer (Zone I)—the *train* signals depict better dispersion trends, whose fundamental modes fit the low velocity of the shallow layer (Vs < 100 m/s). On the other hand, in the same site conditions, the bow and tail signals depict either less clear or higher-phase velocities. This inconsistency might be explained by the higher attenuation induced by the very soft and thicker shallow layer, which is more prominent with fewer energetic bow and tail waves.

The profile in Zone I has a relatively thin and soft soil layer. The most problematic lines (from the viewpoint of track maintenance) have a thicker soft soil layer. Thicker layers might be more detectable but also lead to lower wave speeds, which hinder interpretation. This aspect still has to be evaluated for practical applications.

For the stiffer soil segment (Zone II), the phase velocity spectra appear clearer when using bow wave signals, while for train signals, the energy trend appears a bit more chaotic. To compute S-wave velocity spectra, we selected the phase velocity spectrum with the clearest single-mode and multimode dispersion trends.

The derivation of S-wave profiles is still cumbersome for profiles with relatively soft and thin layers. This clearly needs improvement. This might be based on another interrogator that is able to measure shorter strain gauges more accurately or advanced interpretation methods based on data fusion with, e.g., the results of existing traditional survey methods.

## 8. Conclusions

We demonstrated that fiber-optic telecommunication cables alongside railway tracks together with passing trains as vibration sources can be used to characterize soil stiffness conditions at a high spatial resolution. The frequency response of the recorded acoustic signals generated by train passages can potentially be used to infer the variability of stiffness conditions. Furthermore, the fiber appears to detect the actual driving velocity of the train.

In a thicker soft soil condition, it appears that the best and more consistent phase velocity spectra can be computed when using time windows that contain an actual train passage. On the other hand, for segments with stiffer soil conditions, we determined that the best results can be obtained when using *bow* waves or 30-s time windows just before the train enters the selected fiber segment.

## Data Availability

The data used to support the findings of this study are available from the corresponding authors upon request.
